# 565. Antimicrobial susceptibility profiles of *Acinetobacter* species in blood: 10-year experience from a major United States reference laboratory

**DOI:** 10.1093/ofid/ofad500.634

**Published:** 2023-11-27

**Authors:** Bismarck S Bisono Garcia, Nischal Ranganath, Nicholas Streck, Audrey N Schuetz, Aditya Shah

**Affiliations:** Mayo Clinic, Rochester, Minnesota; Mayo Clinic, Rochester, Minnesota; Mayo Clinic, Rochester, Minnesota; Mayo Clinic, Rochester, Minnesota; Mayo Clinic, Rochester, Minnesota

## Abstract

**Background:**

Antimicrobial resistance (AMR) is a threat to healthcare, and infections with antimicrobial resistant organisms carry significant morbidity and mortality. Owing to high rates of AMR, the Centers for Disease Control and Prevention listed *Acinetobacter baumannii* as a top priority pathogen for research and development of new antimicrobial agents. Antimicrobial susceptibility testing is challenging for this organism, and susceptibility profiles are variable, leading to need of a potential guidance document with assistance in selecting effective empiric therapeutic regimens.

**Methods:**

We retrospectively reviewed the microbiology and antimicrobial susceptibility testing (AST) results for *Acinetobacter* spp. from blood culture isolates submitted to Mayo Clinic Laboratories from December 2012 to 2022. AST was performed using agar dilution and interpretations following Clinical & Laboratory Standards Institute (CLSI) guidelines. Isolates were identified by MALDI mass spectrometry and/or 16S sequencing, as available (Figure 1).

**Figure 1:**
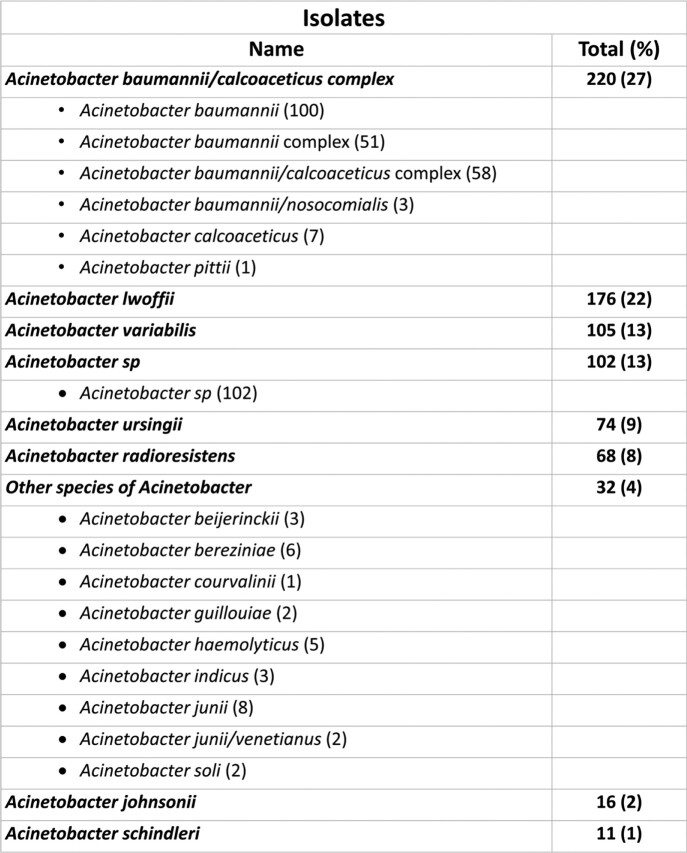
Isolate identification

Isolate identification description. Isolates were analyzed based on this classification used in out laboratory.

**Results:**

804 blood culture isolates were included. *A. baumannii/calcoaceticus* complex (27%) was most frequently isolated, followed by *A. lwoffii* (22%), and showed the highest resistance across all antimicrobials (73% susceptible or below) (Figure 2). *A. baumannii/calcoaceticus* complex showed rising resistance rates over time from 2012 to 2022 for meropenem, minocycline and ampicillin-sulbactam (Figure 3). Cefiderocol non-susceptible rate was 35% for *A. baumannii/calcoaceticus* complex.

**Figure 2:**
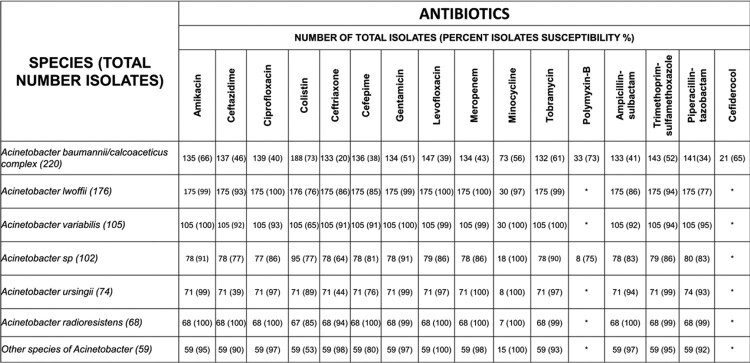
Antimicrobial susceptibility profiles for Acinetobacter spp.

Description of susceptibility profiles for Acinetobacter spp. isolates, based on the classification used by our laboratory. Antimicrobials are presented at the top and isolates on the left. Number of isolates analyzed are presented in the table with the percentage of the isolates susceptible to each antibiotic class in parenthesis.

**Figure 3:**
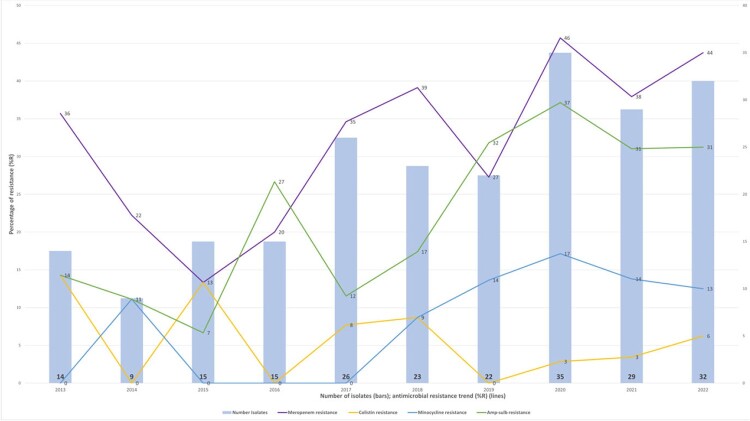
Acinetobacter baumannii/calcoaceticus complex isolates and resistance, trend

Trend of isolates per year and resistance percentage to meropenem, colistin, minocycline and amp-sulb. Number of isolates per year at the base of each bar; percentage of resistance for antimicrobials expressed on lines. Amp-sulb = ampicillin-sulbactam.

**Conclusion:**

*A. baumannii/calcoaceticus* complex showed high levels of resistance ( >75%) across multiple antimicrobials and increasing rates of resistance over time from 2012 to 2022. A high number of cefiderocol non-susceptible organisms is also concerning (Figure 2).

**Disclosures:**

**Audrey N. Schuetz, MD**, Merck: Advisor/Consultant

